# Room-Temperature QCM Sensor Based on GO@WO_3_ Nanocomposites for Ammonia Detection

**DOI:** 10.3390/nano16080467

**Published:** 2026-04-15

**Authors:** Lina Wang, Chong Li, Lei Peng, Junyu Niu

**Affiliations:** 1 School of Electronic Engineering, Huainan Normal University, Huainan 232038, China; wangln@hnnu.edu.cn; 2 Shenzhen Key Laboratory of Advanced Thin Films and Applications, College of Physics and Optoelectronic Engineering, Shenzhen University, Shenzhen 518060, China; 2400231025@mails.szu.edu.cn; 3 Xi’an Structure-Function Materials International Science and Technology Cooperation Base, School of Materials and Chemical Engineering, Xi’an Technological University, Xi’an 710021, China

**Keywords:** quartz crystal microbalance, graphene oxide, WO_3_, quantum dots, NH_3_

## Abstract

The detection of ammonia (NH_3_) at room temperature is of significant importance for environmental monitoring, industrial safety and early disease diagnosis. In this work, a novel room-temperature ammonia sensor was developed by combining graphene oxide with WO_3_ quantum dots. The as-fabricated sensor exhibited excellent comprehensive sensing performance, including high sensitivity, rapid response, outstanding selectivity, and reliable long-term stability. Specifically, when exposed to 10 ppm NH_3_, the sensor based on 1.5% GO@WO_3_ nanocomposites achieved a frequency shift of 578 Hz, which was 6.4 times that of the pure WO_3_ QDs sensor. The theoretical limit of detection (LOD) of the sensor was calculated to be 60 ppb, enabling ppb-level NH_3_ detection. In addition, the sensor demonstrated good long-term stability over a two-week period. The enhanced performance of the GO@WO_3_ nanocomposite sensor is attributed to the formation of an ohmic contact between GO and WO_3_, which eliminates charge transfer barriers, promotes oxygen adsorption, and amplifies the sensing signal. This work provides a simple, efficient, and practical solution for room-temperature NH_3_ detection, offering significant advantages over traditional single-component sensors.

## 1. Introduction

Ammonia (NH_3_) is a highly toxic, corrosive, and flammable gas that plays a critical role in industrial production [1,2]. In industrial processes such as fertilizer synthesis, chemical manufacturing, and food processing, NH_3_ leakage not only causes severe environmental pollution but also poses great threats to the safety of production equipment and on-site operators [3]. Meanwhile, NH_3_ is also an important biomarker for several human diseases [4]. Abnormal concentrations of NH_3_ in exhaled breath are closely associated with chronic kidney disease, liver failure and gastrointestinal disorders [5,6,7]. Consequently, its sensitive and rapid detection is essential for early disease diagnosis. Therefore, the development of high-performance NH_3_ sensors with high sensitivity, fast response, and stable operation at room temperature is urgently needed to meet the practical demands of industrial safety monitoring and clinical disease diagnosis.

In recent years, among various types of gas sensors, quartz crystal microbalance (QCM) sensors have attracted extensive attention in gas detection due to their unique advantages. Compared with traditional resistor gas sensors that require high operating temperatures, QCM sensors operate at room temperature, which effectively reduces energy consumption and avoids the problem of poor stability caused by high temperatures [8]. Additionally, QCM sensors possess high detection accuracy, simple structure, low manufacturing cost, and real-time response characteristics, enabling them to realize trace-level gas detection and be widely applied in environmental monitoring and industrial safety [9,10,11]. These inherent advantages make QCM sensors an ideal platform for room-temperature NH_3_ detection.

On the other hand, the performance of QCM gas sensors is contingent on the sensitive material on the surface of the devices. Therefore, the development and modification of sensitive materials are pivotal to enhancing the gas sensor performance. Tungsten trioxide (WO_3_), as a typical n-type metal oxide semiconductor, has been widely used as a gas-sensitive material due to its excellent chemical stability and high gas adsorption capacity [12,13]. The efficacy of low-dimensional WO_3_ structures, including quantum dots (QDs) [3], nanosheets [14], nanofibers [15], and nanorods [13], in gas detection has been demonstrated. Due to the large surface-to-volume ratio, good solution dispersibility, and tunable bandgap of quantum dots (QDs), their integration with QCM devices holds promise for the development of stable room-temperature gas sensors. However, pure WO3 is subject to inherent limitations, including a narrow detection range and poor selectivity. This complicates the balance of sensitivity, response speed, and stability in practical applications. To address these shortcomings, researchers have focused on low-dimensional material composites. Graphene oxide (GO) is a two-dimensional (2D) carbon nanomaterial with unique structures and properties, including a large specific surface area, abundant functional groups, and high electrical conductivity [16,17]. According to the literature, the overall performance of sensors can be enhanced through the formation of heterojunctions between low-dimensional nanomaterials and 2D graphene oxide (GO), such as SnO_2_/GO [18], TiO_2_/GO [19], MoO_2_/GO [20], ZnO/GO [21], In_2_O_3_/GO [22] and WO_3_/GO [14]. Therefore, the combination of 0D WO_3_ QDs with 2D GO nanosheets to form a 0D/2D nanocomposite structure has the potential to integrate the advantages of both materials, thereby effectively overcoming the performance limitations of single WO_3_ and improving the gas-sensing performance of QCM sensors.

In order to achieve high-precision detection of low-concentration NH_3_, a high-performance room-temperature QCM NH_3_ sensor based on GO@WO_3_ nanocomposites was proposed. The unique quantum-scale structure of WO_3_ and the hierarchical interface between GO and WO_3_ endow the sensor with exceptional adsorption capacity and charge transport efficiency. By rationally optimizing the GO/WO_3_ mass ratio and constructing a uniform heterojunction film, the sensor achieves significantly improved sensitivity, faster response, and better stability compared with pure WO_3_ QDs. The detailed sensing mechanism involving charge transfer and oxygen adsorption behavior is systematically revealed in such a QCM-based NH_3_ sensing system. This study provides a simple and effective strategy for the preparation of high-performance room-temperature NH_3_ sensors and lays a foundation for their practical applications in industrial safety and environmental monitoring.

## 2. Materials and Methods

### 2.1. Chemicals

All reagents are of analytical grade and thus require no further purification before use. The graphene oxide (GO, 99%) was purchased from Macklin Biochemical Technology Co., Ltd. (Shanghai, China). Tungsten chloride (WCl_6_, 99%), oleic acid (OA), ethanol (99%) and oleyl amine (OLA) were supplied by Shanghai Aladdin Biochemical Technology Co., Ltd. (Shanghai, China). The quartz crystal microbalance (QCM) devices with a center frequency of 8 MHz were provided by Shenzhen Jingyuanxing Electronics Co., Ltd. in Shenzhen, China.

### 2.2. Preparation of WO_3_ Quantum Dots and GO@WO_3_ Nanocomposites

**Figure 1:**
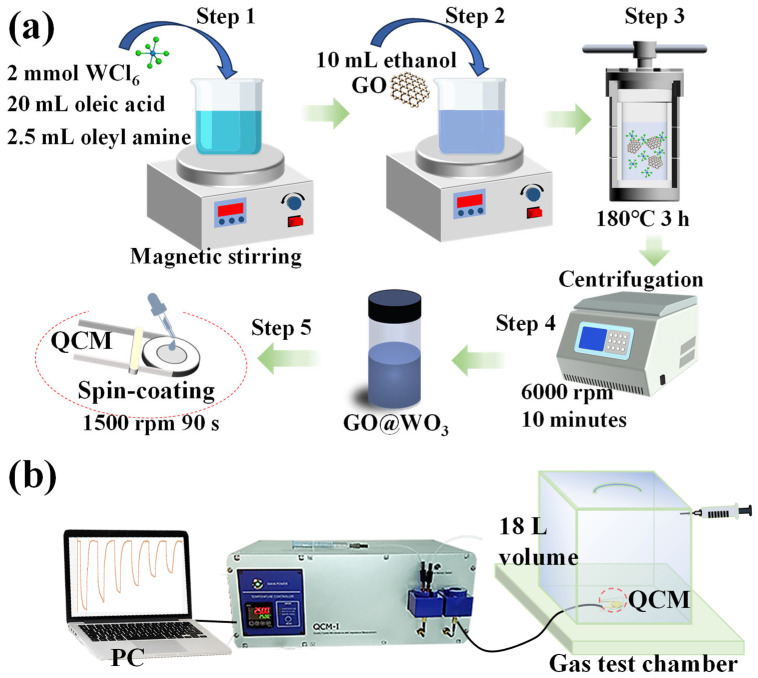
(**a**) The schematics of the synthesis of GO@WO_3_ nanocomposites and the preparation of ammonia QCM sensors. (**b**) The schematics of the device structure for the gas sensing measurements.

The WO_3_ quantum dots were synthesized via the solvothermal method, as previously described in our research [3]. As demonstrated in Figure 1a, 2 mmol of tungsten chloride (WCl_6_) was dissolved in 20 mL of oleic acid (OA) and 2.5 mL of oleyl amine (OLA) at room temperature under magnetic stirring to form a homogeneous precursor solution for the WO_3_ quantum dots. Then, 10 mL of ethanol and GO, with a mass percentage of 0.5% (2.3 mg) relative to WO_3_, were added to the solution. The resulting solution was subsequently added to a Teflon-lined stainless steel autoclave and then subjected to a reaction at a temperature of 180 °C for 3 h. After cooling to room temperature, the obtained 0.5 wt% GO@WO_3_ nanocomposite products were collected by centrifugation at 6000 rpm for 10 min and finally dispersed in a toluene solution at a concentration of 20 mg/mL.

To optimize the sensing performance, a series of GO@WO_3_ nanocomposites with different GO mass percentages (0.5 wt%, 1 wt%, 1.5 wt%, and 2 wt%) were fabricated following the identical procedure, alongside the pure WO_3_ quantum dots as a control sample.

### 2.3. Characterization

The size, microstructure, and surface morphology of the WO_3_ quantum dots, GO, and GO@WO_3_ nanocomposites were investigated using field emission scanning electron microscopy (FE-SEM, Supra 55 Sapphire, Zeiss, Seoul, Republic of Korea) and high-resolution transmission electron microscopy (HR-TEM, FEI Tecnai G2 F20, Thermo Fisher Scientific, Hillsboro, OR, USA). The as-fabricated GO@WO_3_ QCM devices were directly used for FE-SEM testing without sputter coating, as the composite film has excellent intrinsic conductivity, with the QCM chip serving as the substrate. All samples were analyzed for crystalline phases using X-ray diffraction (XRD, MAXima XRD-7000, Shimadzu, Kyoto, Japan) in the 2θ range of 5–80°. The chemical states of the GO@WO_3_ nanocomposite films were studied using an X-ray photoelectron spectrometer (XPS, Escalab 250Xi, Thermo Fisher, Loughborough, UK) and a Fourier transform infrared spectrum (FTIR, Vertex 70, Bruker, Ettlingen, Germany). For the FTIR measurements, the sample was prepared as a thin film spin-coated on a glass substrate, and no KBr pellets were used during the test.

### 2.4. Preparation of Sensors and Gas Sensing Measurement

The QCM device was subjected to sequential ultrasonic cleaning processes involving acetone, alcohol, and deionized water. Subsequently, 20 μL of GO@WO_3_ nanocomposites was deposited onto the QCM device by means of spin coating at a rate of 1500 rpm for 90 s, as illustrated in Figure 1a. The mass of the sensitive material deposited on the QCM device was calculated using the Sauerbrey equation (see Equation (S1)) [23], as outlined in Table S1.

The sensor response was measured using a static sensing system (18 L volume), as illustrated in Figure 1b. The graded concentrations of the target gas were achieved by introducing varied volumes of ammonia (initial concentration: 2%) into the measurement chamber. The operating conditions were established at a room temperature of 24 ± 1 °C and a relative humidity (RH) of 60 ± 1%. The sensor response time and recovery time were defined as the time required to reach 90% of the maximum frequency shift upon exposure to the target gas, and the time required to return to 10% of the frequency shift upon exposure to air, respectively.

## 3. Results

### 3.1. Characterization of Sensitive Materials

**Figure 2:**
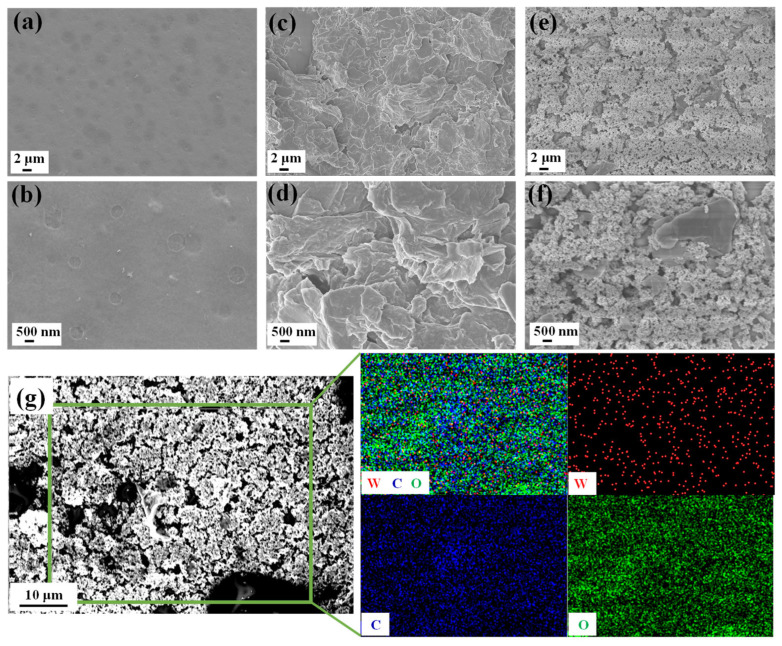
The FE-SEM images of (**a**,**b**) WO_3_, (**c**,**d**) GO and (**e**,**f**) 1.5 wt% GO@WO_3_ nanocomposites. (**g**) The EDS map scanning analysis of W, O and C elements of 1.5 wt% GO@WO_3_ nanocomposites.

The field emission scanning electron microscopy (FE-SEM) morphologies of GO, WO_3_, and GO@WO_3_ nanocomposite films were observed. As illustrated in Figure 2a,b, the images of the WO_3_ film reveal a dense and continuous film structure, which facilitates a stable acquisition of QCM signals. Figure 2c,d indicate that the GO sheets exhibit a typical two-dimensional structure, with the presence of wrinkles providing a substantial number of active sites and spatial configurations for subsequent composite formation. It is worth noting that the GO@WO_3_ nanocomposite effectively formed a fluffy, porous, three-dimensional network structure (see Figure 2e,f). This unique porous structure of GO@WO_3_ nanocomposites provides favorable conditions for enhancing the gas-sensing performance of the QCM sensors. The interconnected porous sensitive film creates a large number of active adsorption sites for target gas molecules, and optimizes the gas diffusion pathways, enabling the gas molecules to efficiently transfer mass to the QCM device [24]. Recent studies have verified that nanocomposites with porous structures enhanced the QCM sensing performance [25,26,27]. The EDS elemental mapping (Figure 2g) results reveal that W, O, and C all exhibit clear and highly uniform distribution patterns, thus confirming the successful formation of the GO@WO_3_ nanocomposite. Furthermore, the absence of discernible component segregation or substantial agglomeration in the mapping verifies the structural uniformity of the sensing layer, which is critical for ensuring a consistent and stable gas-sensing response of the QCM sensor.

**Figure 3:**
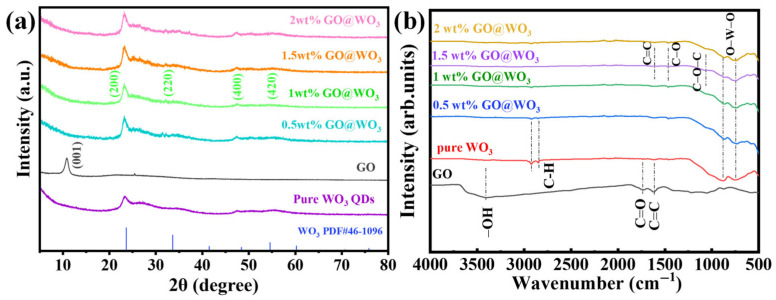
(**a**) The XRD patterns of WO_3_, GO and GO@WO_3_ nanocomposites, and (**b**) the FTIR spectra of GO, WO_3_ and GO@WO_3_ nanocomposites.

Figure 3a shows the XRD patterns of pure WO_3_, GO, and GO@WO_3_ nanocomposite samples at different weight ratios. The diffraction peaks at 23.6°, 33.6°, and 48.4° for pure WO_3_ correspond to the (200), (220), and (400) planes, respectively (PDF#46-1096), confirming the high purity and excellent crystallinity of the synthesized WO_3_. In the XRD pattern of GO, a characteristic peak emerges at 10.4°, corresponding to the [001] plane [28]. All the GO@WO_3_ nanocomposite samples exhibited the primary diffraction peak of WO_3_, with no characteristic diffraction peaks of the GO. This phenomenon can be attributed to the low concentration and uniform distribution of GO within the composite materials [29].

The characterization of the functional groups in GO, pure WO_3_ and the GO@WO_3_ nanocomposites was achieved through the utilization of Fourier transform infrared (FTIR) spectroscopy (see Figure 3b). In the FTIR spectrum of GO, the peaks located at 1732 cm^−1^ and 1625 cm^−1^ correspond to the C=O bond of carbonyl C=C, respectively [30]. The broad band at approximately 3430 cm^−1^ is attributed to the –OH stretching mode of physically adsorbed water molecules [17]. For the GO@WO_3_ nanocomposites, the characteristic peaks at 1073, 1065, and 1634 cm^−1^ correspond to the C-O-C, C-O, and C=C stretching vibrations of GO, respectively [17,28]. In the spectrum of pure WO_3_, the peaks at 748 and 817 cm^−1^ originate from the O–W–O stretching vibrations, while the bands at 2853 and 2923 cm^−1^ are attributed to the aliphatic C–H stretching vibrations of oleic acid (OA) and oleyl amine (OLA) ligands. In comparison with pure WO_3_, the FTIR spectrum of the GO/WO_3_ nanocomposites retains the characteristic peaks of both WO_3_ and GO, while the intensity of the C-H stretching bands (2853–2923 cm^−1^) is significantly weakened. This result indicates that the majority of surface-capped OA and OLA ligands have been removed [23], which is beneficial for the material as it can expose more active sites and facilitate contact with target gas molecules, thereby improving the gas-sensing performance.

**Figure 4:**
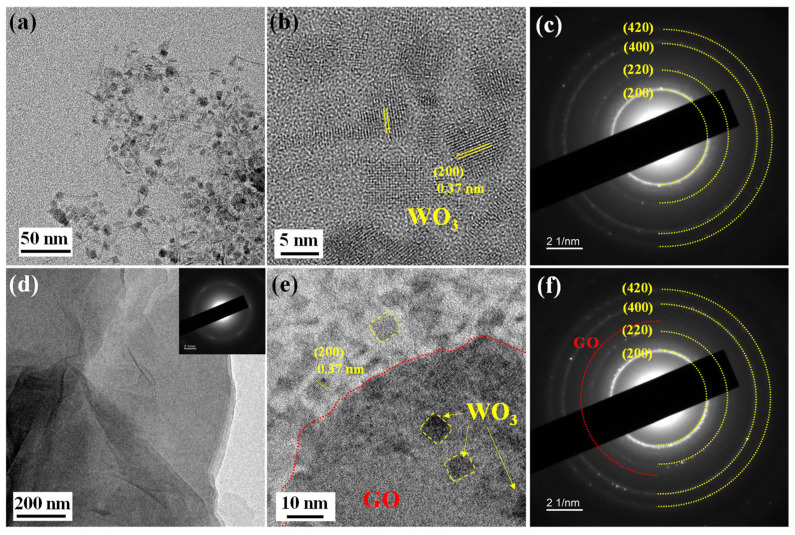
The HR-TEM images of (**a**,**b**) WO_3_ QDs, (**d**) GO and (**e**) 1.5 wt% GO@WO_3_ nanocomposite. Inset of (**d**) is the SAED pattern of GO. The SAED patterns of (**c**) WO_3_ QDs and (**f**) 1.5 wt% GO@WO_3_ nanocomposite.

The microstructural characteristics of pure WO_3_ QDs, GO, and GO@WO_3_ nanocomposites were investigated by high-resolution transmission electron microscopy (HRTEM) and selected area electron diffraction (SAED). As shown in Figure 4a,b, pure WO_3_ QDs exhibit a uniform nanoparticle morphology with particle sizes of 5–10 nm. Figure 4b displays a lattice fringe spacing of 0.37 nm, corresponding to the (200) crystal plane of WO_3_. The corresponding SAED pattern (Figure 4c) reveals the (200), (220), (400), and (420) crystal planes of WO_3_, indicating the polycrystalline nature and good crystallinity of WO_3_ QDs, which corroborates the results from the HRTEM and XRD. GO exhibits typical wrinkled layered morphology (see Figure 4d), and the inserted SAED pattern displays diffuse annular features, indicating that GO possesses a disordered layered structure. As illustrated in Figure 4e, the distribution of WO_3_ QDs on the surface of the GO sheets is consistent and uniform. The 0.37 nm lattice fringes of the WO_3_ (200) crystal plane are clearly observable. Figure 4f displays the characteristic diffraction ring of WO_3_ QDs and the weak diffraction ring of the GO crystal plane, thus confirming the successful formation of nanocomposites of GO and WO_3_ QDs.

**Figure 5:**
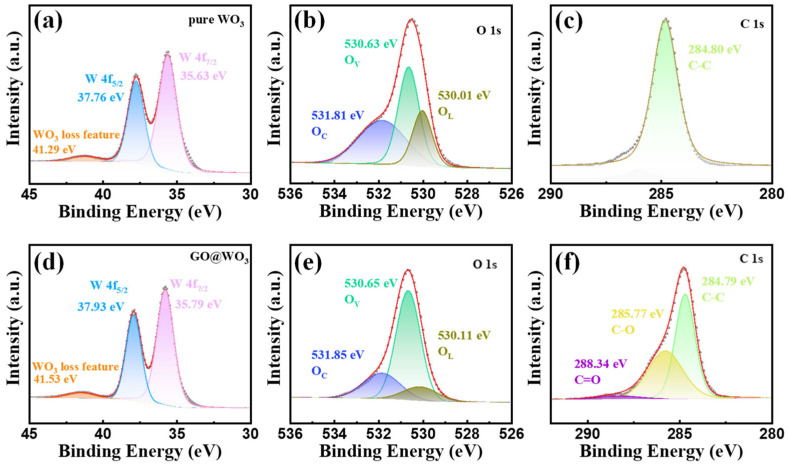
The WO_3_ XPS spectra of (**a**) W 4f, (**b**) O 1s and (**c**) C 1s. A 1.5 wt% GO@WO_3_ nanocomposites XPS spectra of (**d**) W 4f, (**e**) O 1s and (**f**) C 1s.

The surface valence bond states of the WO_3_ QDs and 1.5 wt% GO@WO_3_ nanocomposites were analyzed by XPS, as illustrated in Figure 5. The W 4f spectrum (Figure 5a) displays two characteristic peaks at 37.76 eV and 35.63 eV, corresponding to W 4f_5/2_ and W 4f_7/2_, respectively, which are consistent with the typical valence state of W^6+^ in WO_3_ [31]. An additional satellite peak at 41.29 eV is assigned to the WO_3_ loss feature, further confirming the formation of stoichiometric WO_3_ [32]. The O 1s spectrum (Figure 5b) can be deconvoluted into three components: chemisorbed oxygen (O_C_, 531.81 eV), oxygen vacancies (O_V_, 530.63 eV), and lattice oxygen (O_L_, 530.01 eV). The presence of O_V_ indicates the presence of oxygen vacancy defects in the WO_3_ QDs, which are advantageous for gas adsorption [33]. The C 1s spectrum (Figure 5c) displays a solitary, predominant peak at 284.80 eV, which is ascribed to adventitious carbon originating from the surrounding environment. The W 4f spectrum (Figure 5d) exhibits the characteristic doublet of W 4f_5/2_ (37.93 eV) and W 4f_7/2_ (35.79 eV), with a slight shift to higher binding energy compared to pure WO_3_ QDs. This shift is indicative of a strong electronic interaction between WO_3_ QDs and GO sheets at the heterojunction interface [34]. The O 1s spectrum (Figure 5e) is composed of O_C_ (531.85 eV), O_V_ (530.65 eV), and O_L_ (530.11 eV), with an enhanced O_V_ ratio, suggesting that the introduction of GO further promotes the formation of O_V_ in the composite. The increase in O_V_ indicates that the GO@WO_3_ nanocomposites contain more defect sites compared to the pure WO_3_, which is conducive to gas adsorption and sensor response [35]. Furthermore, the detection of C-C (284.79 eV), C-O (285.77 eV) and C=O (288.34 eV) in the GO@WO_3_ nanocomposites confirms the successful incorporation of GO (see Figure 5f).

### 3.2. Sensing Performance of Sensors

**Figure 6:**
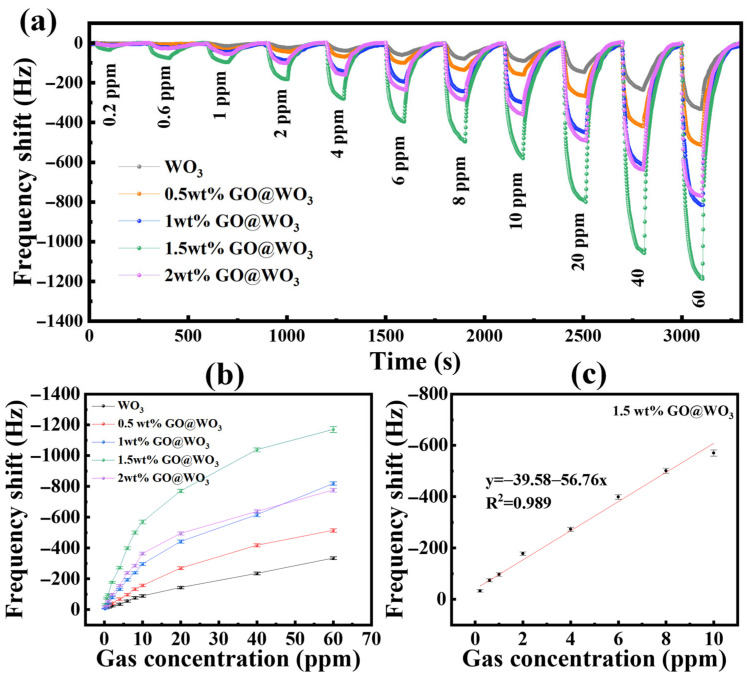
(**a**) The response–time curves with different NH_3_ concentrations based on the WO_3_ sensor and all the GO@WO_3_ sensors. (**b**) The curves between the frequency shift and the gas concentration of all the sensors. (**c**) The frequency shift of the 1.5 wt% GO@WO_3_ sensor to NH_3_ concentrations varying from 200 ppb to 10 ppm.

Figure 6a depicts the real-time frequency response behaviors of THE QCM sensors based on pure WO_3_ QDs and GO@WO_3_ nanocomposites (0.5–2 wt%) under exposure to target gas concentrations ranging from 0.2 ppm to 60 ppm. A pronounced downward frequency shift is observed for all samples upon the introduction of NH_3_ gas, which is attributable to the mass loading effect. The adsorption of NH_3_ molecules onto the sensing layer increases the effective mass of the QCM sensors, resulting in a reduction in resonant frequency. Notably, the incorporation of GO markedly enhances the sensing performance relative to the pure WO_3_ QDs (see Figure 6b). Of all the nanocomposites, the 1.5 wt% GO@WO_3_ sensor has the highest sensitivity. It achieves a frequency shift of −1170 Hz at 60 ppm, which is 3.5 times greater than that of the pure WO_3_ QD sensor (−334 Hz). Even at a concentration of 0.2 ppm, this composite sensor still generates a discernible frequency deviation, demonstrating its capability for low-concentration detection. The improved sensing performance is due to the combined effects of GO and WO_3_ QDs. The large surface area of GO provides many adsorption sites for gas molecules, and its high electrical conductivity promotes interfacial charge transfer. These factors together increase the sensitivity of the QCM sensor. At low concentrations (<10 ppm), the frequency shift increases nearly linearly with gas concentration, indicating the efficient adsorption of gas molecules onto the sensing surface. As the concentration rises beyond 10 ppm, the frequency shift increment rate slows down markedly (Figure 6b). This suggests that the active adsorption sites on the material surface are gradually becoming occupied, which leads to saturation. This saturation behavior arises from the equilibrium between gas adsorption and desorption on the sensing layer. At high concentrations, the adsorption sites become fully occupied, so further increases in gas concentration can no longer induce a proportional increase in frequency shift, resulting in a plateau-like response trend.

Figure 6c shows the linearity analysis of the optimal 1.5 wt% GO@WO_3_ sensor at low concentrations, yielding a high coefficient of determination (R^2^ = 0.989), which confirms excellent linearity in the 0.2–10 ppm range. The theoretical limit of detection (LOD) was calculated to be 60 ppb, demonstrating the sensor’s excellent performance in detecting ammonia at room temperature. The detailed calculation procedure is provided in Equation (S2). Together, these results demonstrate that moderate GO incorporation effectively enhances the sensitivity, linearity and low-concentration detection performance of WO_3_-based QCM gas sensors by providing abundant adsorption sites and facilitating interfacial charge transport.

**Figure 7:**
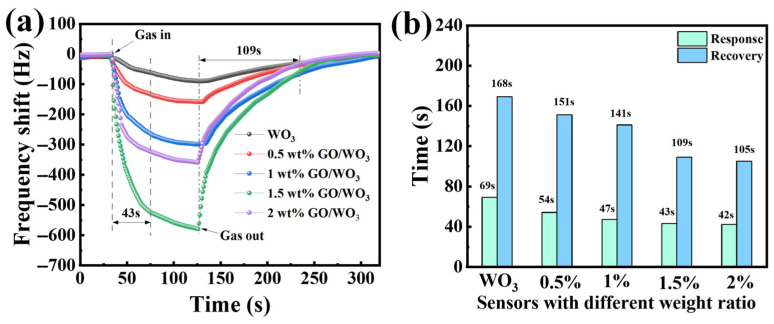
(**a**) The real-time frequency response curve to 10 ppm NH_3_. (**b**) The response and recovery time statistics with 10 ppm NH_3_.

Figure 7a illustrates the real-time dynamic frequency response of pure WO_3_ and GO@WO_3_ nanocomposite sensors upon exposure to 10 ppm NH_3_. All samples exhibit reversible frequency shifts: the frequency decreases rapidly upon the injection of NH_3_ gas and recovers gradually after the gas is removed. As shown in Figure 7b, with increasing GO content, the response time gradually shortens from 69 s (pure WO_3_) to 42 s (2 wt% GO@WO_3_), while the recovery time also decreases from 168 s to 105 s. Notably, the 1.5 wt% GO@WO_3_ sample achieves a balanced optimization of response (43 s) and recovery (109 s) kinetics, demonstrating the best overall dynamic performance. This enhanced response speed is attributed to the high specific surface area and excellent conductivity of GO, which accelerates gas adsorption and interfacial charge transfer.

**Figure 8:**
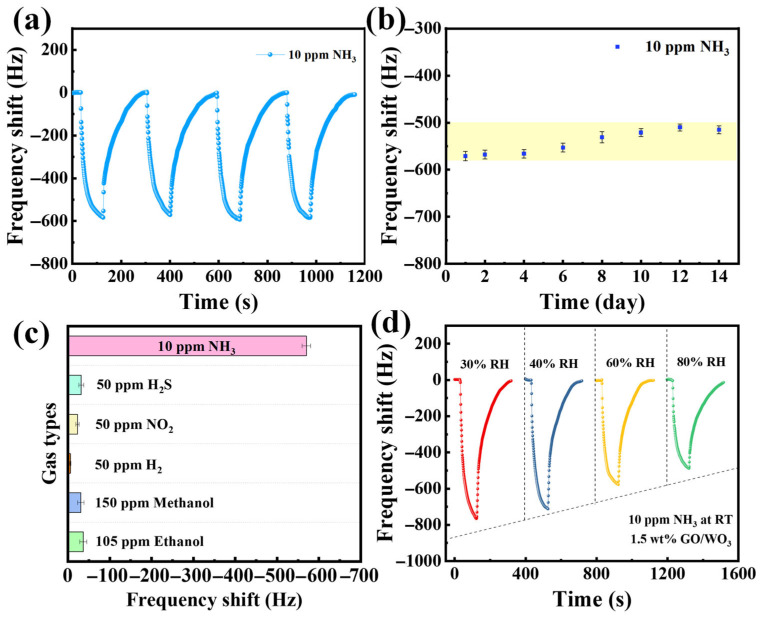
(**a**) The repeated response of the 1.5 wt% GO@WO_3_ sensor to 10 ppm NH_3_. (**b**) The long-term stability of the sensor based on 1.5 wt% GO@WO_3_. (**c**) The selectivity of the sensor based on 1.5 wt% GO@WO_3_. (**d**) The 1.5 wt% GO@WO_3_ sensor response to 10 ppm NH_3_ at different relative humidities.

The 1.5 wt% GO@WO_3_ sensor demonstrates excellent reproducibility and stability towards 10 ppm NH_3_, as shown in Figure 8a. After 14 days, the frequency shift in the 1.5 wt% GO@WO_3_ sensor remained at 90% of its initial response, revealing excellent long-term reliability for practical room-temperature NH_3_ sensing (Figure 8b). The sensor exhibits a much larger frequency shift toward 10 ppm NH_3_ compared to all other interfering gases (even at higher concentrations), demonstrating an excellent selectivity for ammonia detection (Figure 8c). Figure 8d shows the frequency response of the 1.5 wt% GO@WO_3_ sensor to 10 ppm NH_3_ at room temperature across 30–80% RH. Although the response shows slight attenuation with rising humidity, due to water molecules competing for the active sites in the composite, the sensor still exhibits stable, significant frequency shifts even at 80% RH. This confirms its exceptional humidity resistance in practical humid environments.

The performance of the sensor was summarized and compared with that of previously reported WO_3_/carbon-based composite sensors, as shown in Table 1. The WO_3_@GO QCM sensor developed in this work exhibits outstanding overall sensing performance compared with previously reported WO_3_/carbon-based composites. Operating at room temperature, our sensor achieves lower detection limits and faster responses than most WO_3_ sensors modified with carbon materials. These results confirm that combining zero-dimensional WO_3_ quantum dots with two-dimensional GO improves the sensor’s performance.

### 3.3. Sensing Mechanism of the Sensor

**Figure 9:**
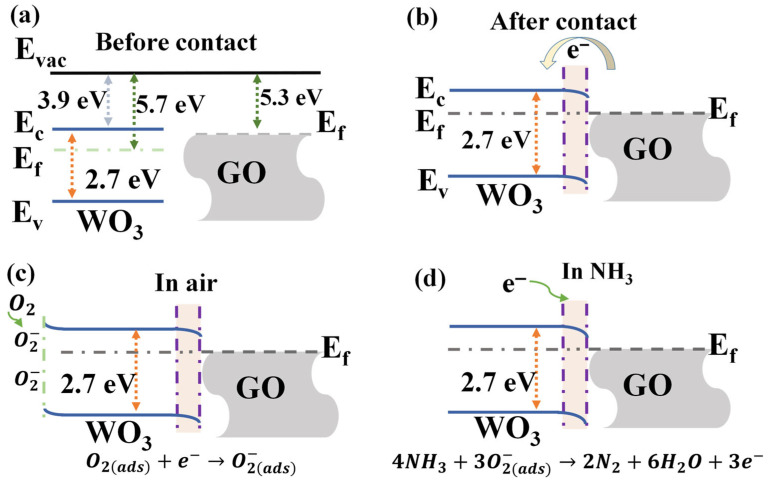
The energy band diagrams of GO and WO_3_ (**a**) before and (**b**) after the contact. The schematic diagram of the NH_3_-sensing mechanism of the sensor based on the GO@WO_3_ nanocomposites (**c**) in the air, and (**d**) exposed to NH_3_.

Figure 9 illustrates the NH_3_-sensing mechanism of the GO@WO_3_ nanocomposites. In the air, ambient O_2_ molecules extract free electrons from the nanocomposites to form ionized adsorbed oxygen species. The type of dominant chemisorbed oxygen species is strongly temperature-dependent: at temperatures below 100 °C, the primary species is $O_{2(ads)}^{-}$; in the intermediate range of 100–300 °C, $O_{\left(ads\right)}^{-}$ becomes the dominant form; and at temperatures exceeding 300 °C, $O_{\left(ads\right)}^{2-}$ is the stable adsorbed oxygen ion [42,43]. In this work, all the sensing measurements were taken at room temperature, thereby confirming $O_{2\left(ads\right)}^{-}$ as the primary active species for the NH_3_ sensing reaction, as shown in Formula (1) [44]. Upon exposure to NH_3_, these adsorbed oxygen ions react with NH_3_ molecules, releasing electrons back into the nanocomposites (Formula (2)) [2,45].

(1) $$O_{2\left(gas\right)}\to O_{2(ads)}^{-}$$

(2) $${4NH}_{3}+{3O}_{2(ads)}^{-}\to {2N}_{2}+{6H}_{2}O+{3e}^{-}$$

The enhanced sensing performance is attributed to the formation of an ohmic contact at the WO_3_/GO interface. As displayed in Figure S1, the current–voltage (I–V) curves of the GO@WO_3_ composite films exhibit good linearity and symmetry within the voltage range of −15 V to +15 V, which is consistent with the characteristic behavior of ohmic contact. To elucidate the charge transfer mechanism at the GO-WO_3_ heterojunction and the gas-sensing behavior, a detailed band alignment diagram is constructed, as shown in Figure 9. It should be noted that the electronic properties of GO, such as its electron mobility, vary with the degree of oxidation, which may have a slight effect on the arrangement of the interface bands [46,47]. WO_3_ exhibits a bandgap of 2.7 eV, an electron affinity of 3.9 eV [15], and a work function of 5.7 eV, while GO has a work function of 5.3 eV. The work function of conductive GO (5.3 eV) is lower than that of n-type WO_3_ (5.7 eV) (Figure 9a) [29]. This results in the migration of electrons from GO to WO_3_ upon contact, thereby bending the energy bands to align their Fermi levels and creating an electron accumulation layer at the heterojunction (Figure 9b). This ohmic contact facilitates an unobstructed charge carrier flow across the interface, while the electron accumulation layer promotes enhanced O_2_ adsorption, leading to the generation of more $O_{2\left(ads\right)}^{-}$ (Figure 9c). The formation of the GO@WO_3_ heterojunction introduces a significant quantity of $O_{2\left(ads\right)}^{-}$, thereby enhancing the adsorption capacity for the NH_3_ gas. As demonstrated in the Sauerbrey equation (see Equation (S1)), this direct mass change on the QCM surface is converted into a measurable frequency shift, which constitutes the gas-sensitive response of the QCM sensor. Upon exposure to NH_3_, the reaction with adsorbed oxygen ions results in the release of electrons. These electrons are then transferred rapidly via an ohmic contact, leading to a reduction in the sensor’s response and recovery times (Figure 9d).

## 4. Conclusions

In summary, a GO@WO_3_ nanocomposite sensor was fabricated for room-temperature NH_3_ detection, exhibiting significantly enhanced sensing performance compared to pure WO_3_. This includes high sensitivity, fast response/recovery speed, excellent selectivity against common interfering gases and long-term stability, with a theoretical limit of detection of 60 ppb for NH_3_ monitoring. The enhanced performance is attributed to the heterojunction formed at the WO_3_/GO interface, which introduces additional adsorbed oxygen molecules that participate in the gas-sensing reaction, thereby improving the response. Furthermore, when exposed to NH_3_, the released electrons are rapidly transferred via ohmic contact, thereby enhancing the sensor’s response speed. This work demonstrates that GO@WO_3_ nanocomposites have the potential to function as effective room-temperature ammonia sensors, thus providing valuable insights into the enhancement of ammonia sensor performance at room temperatures.

## Figures and Tables

**Table 1:** The performance comparison of gas sensors of WO_3_/carbon-based sensors.

Material	Target Gas	Temperature (°C)	Gas Concentration (ppm)	LOD (ppm)	Response/Recovery Time (s)	Reference
WO_3_	NH_3_	142	1.3	-	59/47	[36]
WO_3_/CNTs	NO_2_	RT	1	-	-	[37]
1 wt%CNT/WO_3_ NB	NH_3_	RT	10	-	210/330	[38]
GO/WO_3_ nanorods	NH_3_	200	100	-	10–15	[32]
PPy–GO–WO_3_	NH_3_	RT	10	-	50/120	[30]
rGO/WO_3_ nanowire	NH_3_	300	100	0.138	-	[39]
3% rGO-CuO/WO_3_	Acetone	320	500	1	6/9	[40]
WO_3_/rGO	C_2_H_2_	150	50	1.3	52/27	[41]
WO_3_–N-GO 6%	NO_2_	200	200	-	90/205	[14]
1.5 wt% GO@WO_3_	NH_3_	RT	10	0.06	43/109	This work

## Data Availability

The original contributions presented in this study are included in the article/Supplementary Materials. Further inquiries can be directed to the corresponding authors.

## Supplementary Materials

The following supporting information can be downloaded at https://www.mdpi.com/article/10.3390/nano16080467/s1. Table S1: The preparation parameters of the fabricated sensors, and Table S2: The response values of all samples to different concentrations of ammonia gas. Figure S1. The current-voltage (I–V) characteristics of WO_3_ and GO@WO_3_ nanocomposites.
