# Supplementary material for: Room-Temperature QCM Sensor Based on GO@WO3 Nanocomposites for Ammonia Detection
Source: Nanomaterials (Basel). 2026 Apr 15;16(8):467. doi: 10.3390/nano16080467 (PMC13118550; doi:10.3390/nano16080467)
Supplement: Supplementary file 1 [file nanomaterials-16-00467-s001.zip › nanomaterials-4244777-supplementary.pdf]

Supplementary Information

Room-Temperature QCM Sensor Based on GO@WO_3 Nanocomposites for Ammonia Detection

Lina Wang [1], Chong Li [1,2,*], Lei Peng [2], Junyu Niu [3,*]

1 School of Electronic Engineering, Huainan Normal University, Huainan, 232038, China.

2 Shenzhen Key Laboratory of Advanced Thin Films and Applications, College of Physics and Optoelectronic Engineering, Shenzhen University, Shenzhen 518060, China.

3 Xi'an Structure-Function Materials International Science and Technology Cooperation Base, School of Materials and Chemical Engineering, Xi'an Technological University, Xi'an 710021, Shaanxi, China

* *2170218809@email.szu.edu.cn, niujunyu@st.xatu.edu.cn*

The adsorbed mass on the QCM surface leads to a change of the resonant frequency according to the Sauerbrey equation, as shown in Eq. (S1).

$$\Delta f = \left(-2.26 \times 10^{-6} \frac{f_0^2}{A}\right) \Delta m \qquad \text{(S1)}$$

In the above equation, f_0 is the fundamental resonant frequency of the QCM, Δf is the frequency shift, and A is the surface area of the electrode. Table S1 provides a comprehensive illustration of the fundamental frequencies, frequency shifts and load masses of the sensors.

The limit of detection (LOD) was calculated using the following equation:

$$LOD(ppm) = 3\frac{SD}{|s|} \qquad \text{(S2)}$$

where SD represents the standard deviation of the baseline signal, and s is the slope of the linear fitting curve for frequency shift versus NH_3 concentration. The baseline standard deviation (SD = 1.13 Hz) was obtained from 200 consecutive baseline data points. The slope of the fitting curve in Figure 6c is determined to be |s| = 56.76 $Hz \cdot ppm^{-1}$.

Table S1. Preparation parameters of the fabricated sensors

QCM sensor	Fundamental frequency (Hz)	Frequency shift (Hz)	load mass (ng)
WO_3	7999573	62619	86213
1.5 wt% GO@WO_3 nano-composites	7999631	59354	81675

Table S2. Response values of all samples to different concentrations of ammonia gas.

NH_3 concentration	Response values of WO_3 (Hz)	Response values of 0.5 wt% GO@WO_3 (Hz)	Response values of 1 wt% GO@WO_3 (Hz)	Response values of 1.5 wt% GO@WO_3 (Hz)	Response values of 2 wt% GO@WO_3 (Hz)

0.2 ppm	/	/	-5±1.1	-33±2.1	-15±2.3
0.6 ppm	/	/	-18±1.6	-74±3.1	-24±2.7
1 ppm	-13±1.7	-33±2.3	-39±1.8	-96.8±3.3	-56±3.4
2 ppm	-23±2.3	-41±3.1	-78±3.2	-178±3.2	-98±3.7
4 ppm	-35±3.1	-68±3.4	-132±3.7	-273±3.7	-156±3.9
6 ppm	-56±3.1	-97±3.3	-194±4.1	-399±4.3	-239±4.1
8 ppm	-76±3.4	-133±3.9	-239±4.3	-501±4.5	-286±4.5
10 ppm	-89±3.4	-157±4.1	-296±4.7	-574±4.8	-364±4.7
20 ppm	-143±3.9	-269±4.3	-442±5.1	-770±5.3	-495±5.2
40 ppm	-235±4.1	-418±4.5	-616±5.3	-1038±5.4	-637±5.7
60 ppm	-334±4.6	-514±4.7	-818±6.1	-1170±6.8	-776±6.1

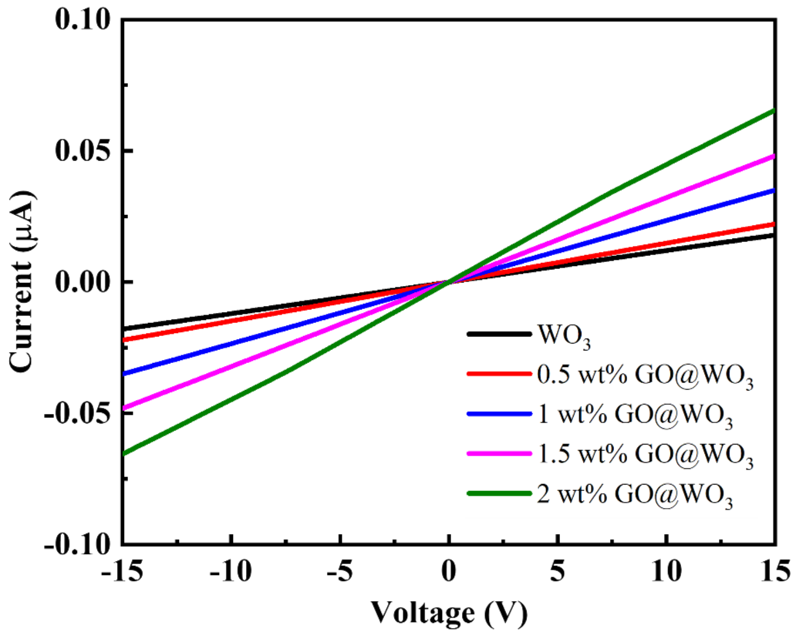

Figure S1. The current-voltage (I-V) characteristics of WO_3 and GO@WO_3 nanocomposites.

For the I-V measurements, the samples were coated onto an alumina ceramic substrate with interdigitated electrodes. The I-V characteristics of the GO@WO_3 nanocomposites were then measured using a Keithley 2400 source meter (Keithley Instruments).
